# Long-term safety and efficacy of darvadstrocel for complex perianal fistulas in Japanese adults with Crohn’s disease: 3-year follow-up results

**DOI:** 10.1093/crocol/otag090

**Published:** 2026-08-18

**Authors:** Tsunekazu Mizushima, Satomi Furukawa, Rikisaburo Sahara, Akihiko Ohta, Yoshiki Okita, Takahiro Ito, Hiroki Ikeuchi, Daijiro Higashi, Akira Sugita, Kenichi Takahashi, Takayuki Ogino, Shigeru Kiryu, Ryo Nakaya, Nobuhiro Narii, Takayoshi Yamaguchi, Kenji Watanabe, Kitaro Futami

**Affiliations:** Department of Colorectal Surgery, Dokkyo Medical University, Mibu, Japan; Department of Coloproctology, JCHO Tokyo Yamate Medical Center, Tokyo, Japan; Department of Proctology, Makita General Hospital, Tokyo, Japan; Department of Coloproctology, Ieda Hospital, Toyota, Japan; Department of Gastrointestinal and Pediatric Surgery, Mie University Graduate School of Medicine, Tsu, Japan; IBD Center, Sapporo Higashi Tokushukai Hospital, Sapporo, Japan; Department of Inflammatory Bowel Disease Surgery, Hyogo Medical University, Hyogo, Japan; Department of Surgery, Fukuoka University Chikushi Hospital, Chikushino, Japan; Department of Inflammatory Bowel Disease, Yokohama Municipal Citizens Hospital, Yokohama, Japan; Inflammatory Bowel Disease Center, Tohoku Rosai Hospital, Sendai, Japan; Department of Gastroenterological Surgery, Graduate School of Medicine, The University of Osaka, Osaka, Japan; Department of Radiology, International University of Health and Welfare Narita Hospital, Narita, Japan; Takeda Development Center Japan, Takeda Pharmaceutical Company Limited, Osaka, Japan; Takeda Development Center Japan, Takeda Pharmaceutical Company Limited, Osaka, Japan; Takeda Development Center Japan, Takeda Pharmaceutical Company Limited, Osaka, Japan; Department of Internal Medicine for Inflammatory Bowel Disease, The University of Toyama, Toyama, Japan; Department of Surgery, Matsunaga Hospital, Fukuoka, Japan

**Keywords:** darvadstrocel, long-term follow-up, mesenchymal stem cell, perianal fistula with Crohn’s disease

## Abstract

**Background/Aims:**

Darvadstrocel is a 1-time local injection for treatment-refractory complex perianal fistulas in patients with Crohn’s disease. Here we present 3-year (week 156) follow-up results from a Japanese phase 3, multicenter, open-label study of darvadstrocel.

**Methods:**

Adult Japanese patients with Crohn’s disease and complex perianal fistulas refractory to previous treatment were treated with darvadstrocel after fistula curettage and seton placement. In this long-term follow-up extension, treatment-emergent adverse events, including serious treatment-emergent adverse events, were recorded through week 156. The proportions of patients with combined remission (clinical remission plus magnetic resonance imaging confirmation of fistula closure) and clinical remission were recorded at week 104 and week 156.

**Results:**

In total, 22 patients were treated with darvadstrocel; 19 patients completed assessment at week 156. Through week 156, treatment-emergent adverse events were experienced by 21/22 patients (95.5%); most treatment-emergent adverse events were moderate (40.9%) or mild (31.8%). Serious treatment-emergent adverse events were reported in 11 patients (50.0%). At week 104 and week 156, 77.3% of patients (17/22) and 68.2% of patients (15/22), respectively, achieved both combined and clinical remission. Mean maximum short axis diameter of the fistula tract at the time of remission was shortest in patients who maintained combined remission until week 156.

**Conclusions:**

No new safety signals for darvadstrocel were identified during the 3-year follow-up period. Combined and clinical remission of complex perianal fistulas was sustained up to 156 weeks.

**ClinicalTrials.gov registration:**

NCT03706456.

## Introduction

Crohn’s disease (CD) is a chronic inflammatory disease of the gastrointestinal tract that occurs predominantly in young people.^1^ Perianal lesions, a common complication of CD, occur with an incidence of 20%-40% worldwide^2^^,^^3^ and 30.3%-58.8% in Asian countries.^1^^,^^4^ In Japan, one study found that at the time of CD diagnosis, approximately half of patients have perianal lesions, with perianal fistulas and/or abscesses being the most common lesions^5^; another found that nearly 70% of patients with perianal fistula with CD had their fistulas diagnosed before diagnosis with CD.^6^ Approximately, half of Japanese patients with perianal fistulas are classified as having complex perianal fistulas, for example, with multiple external openings, associated with perianal abscess, etc.^7^ Common symptoms of perianal fistulas include chronic anal pain, perianal itching, fistula discharge, and fecal incontinence, which can lead to frequent admissions to hospital, reduction in work productivity, and impairment of daily activities.^5^^,^^8^^,^^9^ This symptom burden also has a negative effect on patients’ quality of life (QoL).^3^^,^^8^^,^^9^

Treatment options for perianal fistulas with CD include medical and surgical therapies, and stoma creation and proctectomy may be considered for severe cases.^10–12^ For complex perianal fistulas, conventional fistulotomy presents a high risk of relapse, impaired wound healing, and damage to the anal sphincter muscle.^10^^,^^12^ Seton drainage in combination with antibiotics is a common initial treatment strategy for complex perianal fistulas^7^^,^^10^^,^^11^; however, seton drainage is not a curative treatment for fistula healing^7^ and may lead to a negative impact on patients’ sense of well-being.^13^ As the current standard treatment, surgical therapy in combination with anti-human tumor necrosis factor-α (TNFα) monoclonal antibodies, such as infliximab, has been recommended for the induction and maintenance of remission in complex perianal fistulas with CD in the 2024 European Crohn’s and Colitis Organisation Guidelines.^12^ However, fistula closure rates are not high with infliximab treatment, with 19%-36% of patients achieving complete fistula closure within 1 year in clinical trials.^14^ More recent studies of the Janus kinase inhibitor upadacitinib have similarly demonstrated low fistula closure rates (16.0%-18.8%) within 1 year of treatment.^15^ Therefore, effective management and long-term healing of treatment-refractory complex perianal fistulas with CD can be difficult to achieve.

Darvadstrocel is a suspension of expanded human allogeneic mesenchymal stem cells extracted from adipose tissue developed as a 1-time local injection for the treatment of treatment-refractory complex perianal fistulas in patients with CD.^16–18^ Approval of darvadstrocel in Japan was on the basis of results from the pivotal ADMIRE-CD clinical trial that demonstrated efficacy and safety in non-Japanese patients,^16^^,^^17^ and also the Japanese phase 3 study.^19^ In the Japanese phase 3 study, no new safety findings were identified, and combined remission (clinical remission together with magnetic resonance imaging [MRI] confirmation) was achieved by 59.1% of patients at week 24 and 68.2% of patients at week 52.^19^ There is evidence supporting the sustained long-term safety and effectiveness of darvadstrocel, from the follow-up extension of the ADMIRE-CD study^20^ and from the latest systematic review and meta-analysis of observational studies of darvadstrocel.^18^ However, recent results from the ADMIRE-CD-II randomized controlled trial did not show superiority of darvadstrocel over placebo (combined remission rate of darvadstrocel vs placebo at week 24: 48.8% vs 46.3%, and at week 52: 41.0% vs 39.7%).^21^ The safety profile of darvadstrocel in ADMIRE-CD-II was consistent with previous clinical trials.^21^

In this article, we present the results of the 3-year follow-up extension of the Japanese phase 3, multicenter, open-label study of darvadstrocel. The objectives of this long-term extension study were to evaluate the safety of darvadstrocel for the treatment of perianal fistulas in adult patients with CD in Japan over 156 weeks and to assess efficacy at week 104 and week 156.

## Methods

### Study design and study population

This was a phase 3, multicenter, open-label study of darvadstrocel for the treatment of complex perianal fistulas in adult Japanese patients with CD, conducted from March 2019 to February 2023. Following the marketing approval of darvadstrocel in Japan in 2021, the study was switched to a post-marketing clinical trial. The study was conducted according to the principles of the Declaration of Helsinki and was approved by the local or central Institutional Review Boards of all the study sites. All patients provided informed consent. The study was registered with ClinicalTrials.gov (NCT03706456).

Full details of the study design and patient eligibility criteria have been published previously.^19^ In brief, eligible patients were aged ≥18 years with (1) non-active or mildly active CD, as defined by a Crohn’s Disease Activity Index (CDAI) score ≤220 (evaluated at any time in the screening period) for at least 6 months; (2) complex perianal fistula(s) with a maximum of 2 internal openings and a maximum of 3 external openings, confirmed by clinical assessment and MRI; and (3) fistula(s) that had been draining for at least 6 weeks prior to the screening. Patients were excluded if they (1) had concomitant rectovaginal or rectourethral or bladder fistula(s), rectal or anal stenosis, active proctitis, diverting stomas, or an abscess or collection >2 cm in diameter; (2) had undergone surgery other than drainage or seton placement for the active fistula; (3) were naïve to previous treatments for complex perianal fistulizing CD (ie, antibiotics, immunosuppressants, or biologics); and/or (4) had received systemic steroids in the previous 4 weeks.

Complex perianal fistulas were defined as meeting 1 or more of the following criteria: (1) high (ie, above the dentate line) inter-sphincteric or trans-sphincteric fistula, extra-sphincteric fistula, or supra-sphincteric fistula; (2) presence of ≥2 external openings (tracts); and/or (3) associated fluid collections. Patients were included in the study if their complex perianal fistulas had been previously treated and refractory (inadequate response, loss of response, or intolerance) to at least 1 of the following treatments: antibiotics, immunosuppressants, or biologics (anti-TNFs, anti-integrin or anti-interleukin-12/23). The study permitted the continuation of the baseline treatment for CD (ie, biologics, immunosuppressants, etc. that were in use during the screening period) in an add-on design. Although there were no particular restrictions for medications and treatments after week 52, patients were asked to follow the rules for allowed and excluded medications set before week 52^19^ as far as possible.

### Procedures

As described previously,^19^ patients’ eligibility was assessed at a screening visit (Visit 1). Baseline MRI of fistulas was performed between Visit 1 and Visit 2. Eligible patients received fistula curettage and seton placement under anesthesia at the preparation visit (Visit 2; day −21 to −14). Seton(s) were removed just prior to the study product administration at day 1 (Visit 3), and 24 mL of darvadstrocel, a cell suspension containing 120 × 10^6^ cells of expanded allogeneic adipose-derived stem cells (Cx601), was administered to all patients as a single intralesional dose on day 1. Follow-up visits for the clinical assessments, including fistula closure, occurred at weeks 2, 4, 8, 16, 24, 40, 52, 104, and 156. MRI assessments of fistula closure were performed at weeks 24, 52, 104, and 156.

### Safety endpoints

Safety endpoints were treatment-emergent adverse events (TEAEs), including serious TEAEs, through week 156. TEAEs were defined as any adverse event (AE) whose date of onset occurred on or after receiving the study treatment. TEAEs were coded using the Medical Dictionary for Regulatory Activities (MedDRA) version 25.0 and described using MedDRA system organ class and preferred terms.

### Efficacy endpoints

#### Primary efficacy endpoint

The primary efficacy endpoint was the proportion of patients with combined remission at week 104 and week 156. Combined remission was defined as the clinically confirmed closure of all treated external openings (ie, absence of drainage when gentle finger compression was applied) and absence of collections >2 cm in the treated fistulas, confirmed by central MRI assessment.

#### Secondary efficacy endpoints

Secondary efficacy endpoints included the following: the proportion of patients with clinical remission at week 104 and week 156 (where clinical remission was defined as the clinically confirmed closure of all treated external openings); the proportion of patients with relapse at week 52 in patients with combined remission at week 24; the proportion of patients with relapse at week 104 and week 156 in patients with combined remission at week 52; and Perianal Disease Activity Index (PDAI) score, CDAI, and Van Assche score up to week 156. Relapse was defined as the clinically confirmed reopening of any of the treated external openings with active drainage, or the development of a collection >2 cm in the treated fistulas, confirmed by central MRI assessment.

#### Exploratory efficacy endpoints

Exploratory efficacy endpoints included the following: relationships between combined remission at week 104 and week 156 and patients’ sex, age, and number of internal/external openings; the association of fistula healing with measurements obtained from MRI; patients’ medical treatment (biologics, immunosuppressants, combination of biologics and immunosuppressants, or neither) at week 156 and the change in medical treatment from after week 52 to week 156 and their relationships with remission status at week 156 (combined remission or non-remission); and the proportion of patients in remission and non-remission at each evaluation visit.

Measurements obtained from MRI were the distance between internal opening(s) and the anal verge, measured at baseline; length of fistula tracts (distance between internal and external openings), measured at baseline; separation of internal and external sphincters, measured at baseline; and the maximum short axis diameter of fistula tracts, measured at baseline, when remission was achieved and at any relapse time points. The distance between the anal verge and the internal opening was measured after identifying the anal verge on sagittal or coronal images. Fistula tract lengths were measured as the distance between internal and external openings in the imaging plane most parallel to the course of the tract; for curved fistulas, measurements were made using 2 or more planes. The separation of internal and external sphincters was evaluated using 1 image, sliced at the middle position between the upper and lower anal canals, and was scored as score 1, 2, or 3. Score 1 indicated that the internal and external sphincters were separated almost around the entire circumference of the anal canal, score 3 indicated that it was almost impossible to separate the entire circumference of the anal canal, and score 2 indicated a situation between score 1 and score 3. The maximum short axis diameter was defined as the short axis diameter of the fistula tract on the plane that showed the maximum diameter among all the collected imaging planes. These data were collated for patients who achieved combined remission and maintained remission through week 156, those who relapsed during the 156-week period, and those who did not achieve remission during the 156-week period. An example measurement of fistula tract length is shown in Figure S1.

### Statistical analysis

Safety analyses were performed on the safety analysis set, which consisted of all patients who received the study treatment. Efficacy analyses were performed on the intent-to-treat population, which consisted of all patients who enrolled into the treatment period. The proportions of patients with combined remission and clinical remission are presented as the percentage of patients, with 2-sided 95% confidence intervals. Length of fistula tract and maximum short diameter of fistula tracts (from MRI) were measured in mm, and data are presented as mean (SD). Subgroup analyses were conducted to describe relationships between combined remission at week 104 and week 156 and patient sex, age, and number of internal/external openings. All analyses were performed using SAS software, version 9.4 (SAS Institute Inc., Cary, NC, USA).

## Results

### Study population

A total of 26 patients signed the informed consent form (Figure 1). Four patients were ineligible for the study product administration due to screen failure (3 patients) and voluntary withdrawal (1 patient). A total of 22 patients were treated with darvadstrocel, and 19 patients (86.4%) completed the study assessment at week 156. Three patients (13.6%) prematurely discontinued the study before week 156. Two patients discontinued the study before week 52 due to lack of efficacy, and 1 patient discontinued the study during the long-term follow-up period (from week 52 to week 156) due to an AE. The mean (SD) duration of follow-up after baseline was 143.7 (38.57) weeks.

**Figure 1:**
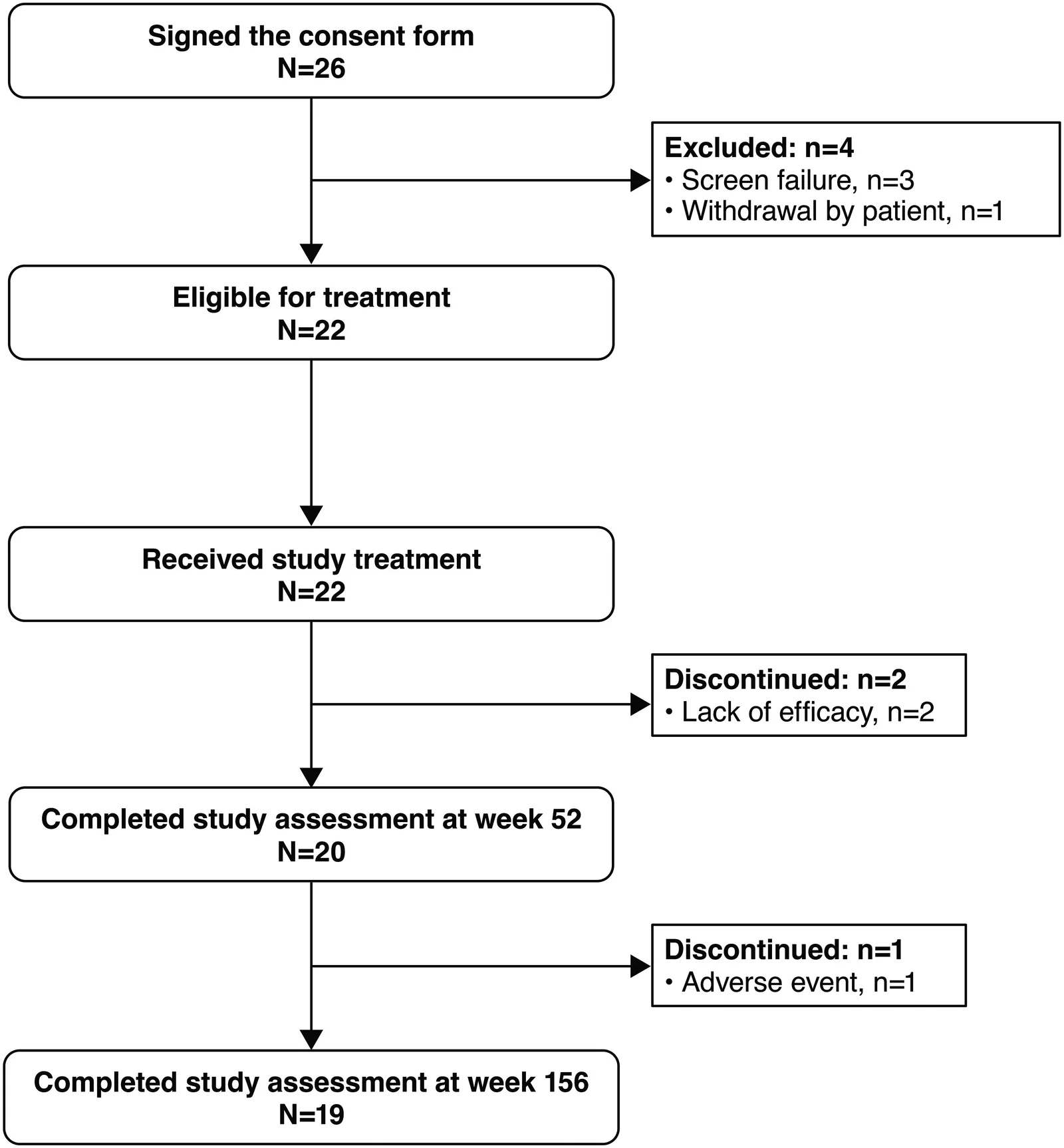
Patient disposition flow chart.

### Demographic and baseline clinical characteristics

Full details of the baseline demographics have been published previously.^19^ In brief, 63.6% of patients were male, the mean (SD) age of the patients was 36.4 (10.36) years, and mean (SD) duration of CD was 11.3 (6.64) years (Table 1). All patients received concomitant medication at baseline. Nine patients (40.9%) received a single biologic only (infliximab only, *n* = 6; adalimumab only, *n* = 1; ustekinumab only, *n* = 2). Two patients (9.1%) received immunosuppressants only (azathioprine). Seven patients (31.8%) received both biologics and immunosuppressants (infliximab + azathioprine, *n* = 5; adalimumab + azathioprine, *n* = 2). Most patients (72.7%) had a fistula with 1 internal opening, whereas the remaining patients (27.3%) had 2 internal openings. The proportions of patients who had 1, 2, and 3 external openings were 13.6%, 63.6%, and 22.7%, respectively. The most common distribution of topography of internal openings and external openings was 1 internal opening and 2 external openings (50.0% of patients).

**Table 1:** Baseline demographics and clinical characteristics (ITT population).

Variable	Patients (*N* = 22)
**Age, years**	
** Mean (SD)^a^**	36.4 (10.36)
** Median (Q1, Q3)**	34.5 (29.0, 41.0)
** Minimum**	19
** Maximum**	62
**Sex, male, *n* (%)^a^**	14 (63.6)
**Duration of CD, years**	
** Mean (SD)^a^**	11.3 (6.64)
** Median (Q1, Q3)**	9.7 (6.4, 17.5)
** Minimum**	1
** Maximum**	21
**Concomitant medication, *n* (%)^a^**	
** Biologics only**	9 (40.9)
** Immunosuppressants only**	2 (9.1)
** Biologics + immunosuppressants**	7 (31.8)
** Neither**	4 (18.2)
**Topography of internal and external openings at baseline, *n* (%)^a^**	
** 1 internal opening**	16 (72.7)
** 2 internal openings**	6 (27.3)
** 1 external opening**	3 (13.6)
** 2 external openings**	14 (63.6)
** 3 external openings**	5 (22.7)
** 1 internal opening + 1 external opening**	3 (13.6)
** 1 internal opening + 2 external openings**	11 (50.0)
** 1 internal opening + 3 external openings**	2 (9.1)
** 2 internal openings + 1 external opening**	0 (0.0)
** 2 internal openings + 2 external openings**	3 (13.6)
** 2 internal openings + 3 external openings**	3 (13.6)
**Topographic of draining external openings at screening, *n* (%)**	
** 1 external opening**	3 (13.6)
** 2 external openings**	15 (68.2)
** 3 external openings**	4 (18.2)

Abbreviations: CD, Crohn’s disease; ITT, intent-to-treat; Q1/3, Quartile 1/3.

a Previously reported (Furukawa et al.^19^).

### Safety outcomes through week 156

Safety data through week 52 have been reported previously.^19^ Through week 156, TEAEs were experienced by 21/22 patients (95.5%); most TEAEs were moderate (40.9%) or mild (31.8%) (Table 2). Among these patients, 2/22 patients (9.1%) were judged to have experienced treatment-related TEAEs; as previously reported, these treatment-related TEAEs occurred during the first 52 weeks and were CD and diarrhea in 1 patient, and blood bilirubin increased in 1 patient.^19^ One patient had a TEAE that led to study discontinuation; this occurred after week 52, and the event was a relapse of perianal fistula. No deaths, no anorectal cancer, and no other TEAEs of special interest occurred through week 156.

**Table 2:** TEAEs through week 156, including serious TEAEs and TEAEs that occurred in ≥2 patients (Safety Analysis Set).

	Patients, *n* (%) (*N* = 22)	
	Weeks 1 to 156	After week 52
**TEAEs**	21 (95.5)	17 (77.3)
** Treatment-related TEAEs**	2 (9.1)	0 (0.0)
** Mild**	7 (31.8)	6 (27.3)
** Moderate**	9 (40.9)	8 (36.4)
** Severe**	5 (22.7)	3 (13.6)
** Leading to study discontinuation**	1 (4.5)	1 (4.5)
**Serious TEAEs**	11 (50.0)	9 (40.9)
** Related to treatment**	1 (4.5)	0 (0.0)
** Leading to study discontinuation**	0 (0.0)	0 (0.0)
**Deaths**	0 (0.0)	0 (0.0)

**Serious TEAEs**		
**System organ class Preferred term** ^a^		

**Gastrointestinal disorders**	6 (27.3)	4 (18.2)
** Crohn’s disease** ^b^	3 (13.6)	2 (9.1)
** Perianal fistula** ^c^	1 (4.5)	1 (4.5)
** Intestinal obstruction**	1 (4.5)	0 (0.0)
** Small intestinal obstruction**	1 (4.5)	1 (4.5)
**Infections and infestations**	4 (18.2)	4 (18.2)
** COVID-19**	4 (18.2)	4 (18.2)
**Injury, poisoning, and procedural complications**	1 (4.5)	0 (0.0)
** Intestinal anastomosis complication**	1 (4.5)	0 (0.0)
**Pregnancy, puerperium and perinatal conditions**	1 (4.5)	1 (4.5)
** Premature labor**	1 (4.5)	1 (4.5)
**Renal and urinary disorders**	2 (9.1)	0 (0.0)
** Calculus urinary**	1 (4.5)	0 (0.0)
** Tubulointerstitial nephritis**	1 (4.5)	0 (0.0)

**TEAEs that occurred in ≥2 patients**		
**System organ class Preferred term^a^**		

**Gastrointestinal disorders**	18 (81.8)	13 (59.1)
** Perianal fistula** ^c^	7 (31.8)	5 (22.7)
** Proctalgia**	7 (31.8)	1 (4.5)
** Crohn’s disease** ^b^	4 (18.2)	2 (9.1)
** Cheilitis**	2 (9.1)	2 (9.1)
** Dental caries**	2 (9.1)	1 (4.5)
** Nausea**	2 (9.1)	1 (4.5)
**General disorders and administration site conditions**	3 (13.6)	2 (9.1)
** Pyrexia**	2 (9.1)	1 (4.5)
**Infections and infestations**	16 (72.7)	8 (36.4)
** Nasopharyngitis**	6 (27.3)	1 (4.5)
** COVID-19**	4 (18.2)	4 (18.2)
** Pharyngitis**	2 (9.1)	1 (4.5)
** Rhinitis**	2 (9.1)	1 (4.5)
**Musculoskeletal and connective tissue disorders**	8 (36.4)	5 (22.7)
** Back pain**	3 (13.6)	2 (9.1)
** Fistula**	2 (9.1)	1 (4.5)
**Renal and urinary disorders**	4 (18.2)	0 (0.0)
** Calculus urinary**	2 (9.1)	0 (0.0)
**Neoplasms benign, malignant and unspecified**	2 (9.1)	1 (4.5)
** Acrochordon**	2 (9.1)	1 (4.5)

Abbreviations: COVID-19, coronavirus disease 2019; MedDRA, Medical Dictionary for Regulatory Activities; TEAE, treatment-emergent adverse event.

a MedDRA (version 25.0) preferred term.

b Indicates worsening of Crohn’s disease.

c MedDRA (version 25.0) preferred term is “anal fistula,” but we have used “perianal fistula” throughout for consistency.

Up to week 156, serious TEAEs were reported in 11 patients (50.0%), and a treatment-related serious TEAE was reported in 1 patient (Table 2). As previously reported, the treatment-related serious TEAE was CD and was observed during the first 52 weeks of the study.^19^ Serious TEAEs occurring after week 52 included COVID-19 (4 patients, 18.2%), worsening of CD (2 patients, 9.1%), perianal fistula (1 patient, 4.5%), small intestinal obstruction (1 patient, 4.5%), and premature labor (1 patient, 4.5%). Other serious TEAEs, which occurred during the first 52 weeks of the study, were intestinal obstruction, intestinal anastomosis complication, calculus urinary, and tubulointerstitial nephritis.^19^

TEAEs that occurred in ≥2 patients through week 156 are listed in Table 2. TEAEs occurring in >5 patients were perianal fistula (31.8%, 7/22 patients), proctalgia (31.8%, 7/22 patients), and nasopharyngitis (27.3%, 6/22 patients). TEAEs that occurred in ≥2 patients after week 52 were perianal fistula in 5 patients (22.7%), COVID-19 in 4 patients (18.2%), and worsening of CD, cheilitis, and back pain in 2 patients each (9.1% each).

There were 9 events in 7 patients of perianal fistula reported as TEAEs through week 156, including 5 events in 4 patients of worsening of treated perianal fistula(s), and 4 events in 4 patients of new perianal fistulas. One patient had worsening of the treated perianal fistula and new perianal fistula.

### Efficacy outcomes through week 156

#### Primary and secondary endpoints

At week 104 and week 156, 77.3% of patients (17/22) and 68.2% of patients (15/22), respectively, achieved combined remission (Figure 2A). The proportions of patients achieving clinical remission at week 104 and week 156 were 77.3% (17/22 patients) and 68.2% (15/22 patients), respectively (Figure 2B). Among patients who achieved combined remission at week 52, none relapsed at week 104, and 20.0% (3/15) experienced relapse at week 156 (Figure 2C).

**Figure 2:**
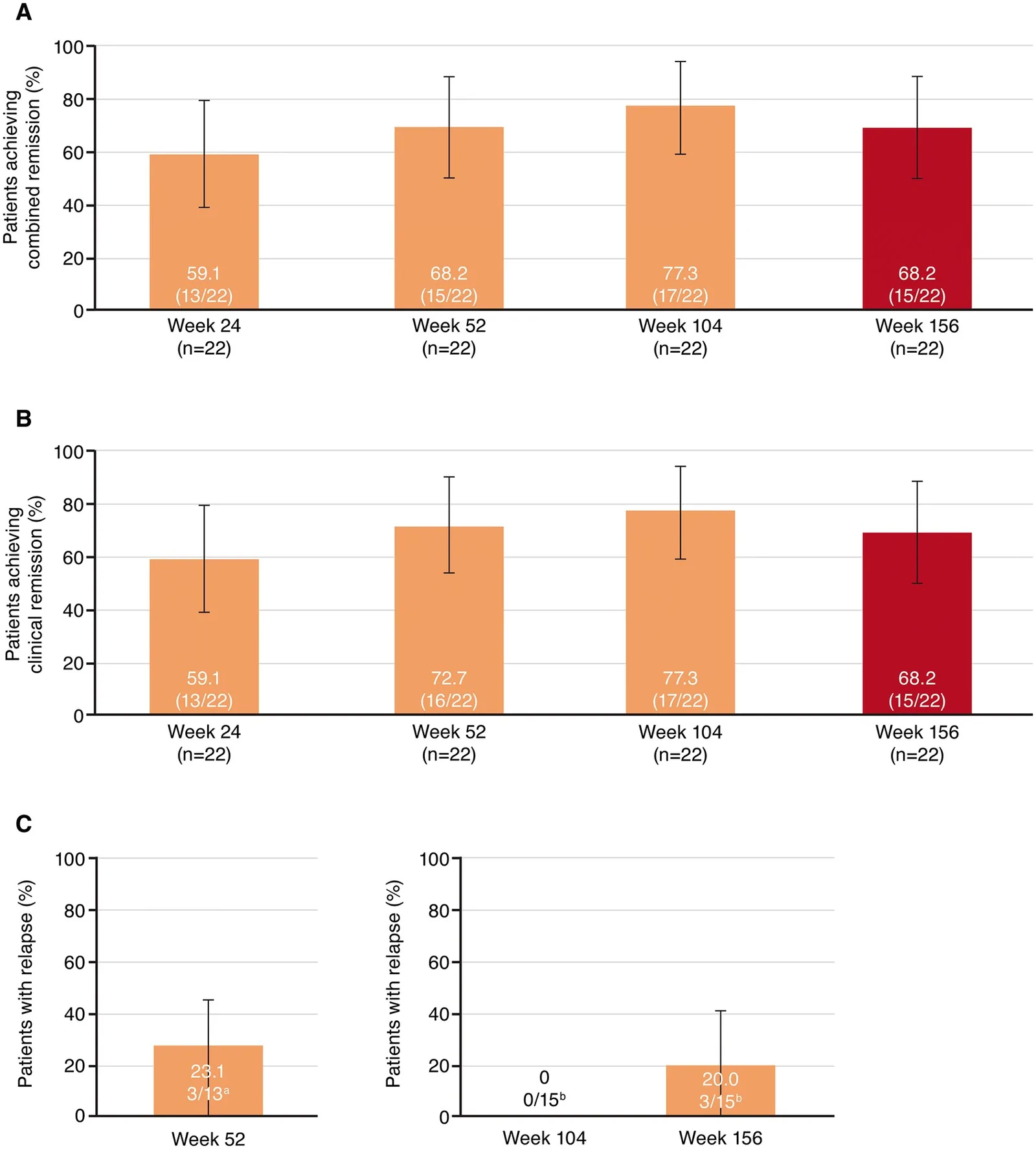
(A) Percentage of patients achieving combined remission through week 156. Error bars indicate 95% CIs. Combined remission was defined as absence of drainage when gentle finger compression was applied to all treated external openings and absence of collections >2 cm on MRI. (B) Percentage of patients achieving clinical remission through week 156. Error bars indicate 95% CIs. Clinical remission was defined as clinically confirmed closure of all treated external openings without drainage when gentle finger compression was applied. (C) Relapse at weeks 52, 104, and 156 in patients with earlier combined remission. Relapse was defined as the clinically confirmed reopening of any of the treated external openings with active drainage, or the development of a collection >2 cm in the treated fistulas confirmed by central MRI assessment. ^a^Number of patients who achieved combined remission at week 24. ^b^Number of patients who achieved combined remission at week 52. CI, confidence interval; MRI, magnetic resonance imaging.

Mean PDAI scores decreased over time compared with baseline over 156 weeks, and individual domain scores followed the same trend (Table 3). The mean (SD) change from baseline to week 156 in PDAI total score was −3.2 (1.61), in CDAI score was −21.11 (53.727), and in Van Assche total score was −3.8 (5.01).

**Table 3:** Disease activity through week 156 (ITT population).

Week	PDAI total score		CDAI score		Van Assche total score	
	Value at visit	Change from baseline	Value at visit	Change from baseline	Value at visit	Change from baseline
**Baseline**	*N* = 22		*N* = 22		*N* = 18	
** Mean (SD)**	4.8 (2.15)		94.25 (60.048)		14.5 (3.99)	
**Week 52**	*N* = 20		*N* = 21		*N* = 21	*N* = 17
** Mean (SD)**	2.1 (2.11)	−2.8 (2.59)	73.66 (73.612)	−20.86 (51.331)	11.2 (5.34)	−2.2 (4.90)
**Week 104**	*N* = 20					
** Mean (SD)**	1.9 (1.95)	−3.0 (2.00)	–	–	–	–
**Week 156**	*N* = 20		*N* = 20		*N* = 20	*N* = 16
** Mean (SD)**	1.7 (1.95)	−3.2 (1.61)	71.44 (51.202)	−21.11 (53.727)	10.4 (5.42)	−3.8 (5.01)

Abbreviations: CDAI, Crohn’s Disease Activity Index; ITT, intent-to-treat; PDAI, Perianal Disease Activity Index.

#### Exploratory endpoints

Subgroup analysis assessing the impact of age, sex, and the number of internal/external openings on combined remission at week 104 and week 156 showed that none of these factors affected combined remission (Table S1).

To explore potential predictors of remission and relapse, several measurements were made retrospectively from MRI. The mean (SD) distance between internal opening and the anal verge was longest in patients who relapsed (26.73 [14.62] mm for all fistula tracts in these patients) (Table 4). The mean (SD) length of the fistula tract at baseline was shortest in patients who maintained combined remission until week 156 after achieving combined remission (39.25 [17.41] mm), followed by patients who did not achieve remission during the 156-week period (42.50 [14.81] mm), and those who relapsed during the 156-week period (51.92 [32.67] mm for all fistula tracts in these patients and 56.18 [42.83] mm for only the relapsed fistula tracts). The mean (SD) score for the separation of internal and external sphincters was highest in patients who relapsed (2.17 [0.75]).

**Table 4:** Relationships between remission, relapse, and MRI image measurements.

	Patients who maintained combined remission until week 156 after achieving combined remission (*N* = 12)^a^	Patients who relapsed during the 156-week period (*N* = 6)		Patients who did not achieve remission during the 156-week period (*N* = 4)^c^
		All fistula tracts	Relapsed fistula tracts^b^	
**Distance between the internal opening and the anal verge (mm)**	23.51 (11.57)	26.73 (14.62)	24.88 (15.09)	17.35 (4.74)
**Length of fistula tract at baseline (distance between internal and external openings, mm)**	39.25 (17.41)	51.92 (32.67)	56.18 (42.83)	42.50 (14.81)
**Separation of internal and external sphincters (score 1-3)**	2.00 (0.95)	2.17 (0.75)	2.17 (0.75)	1.00 (0.00)
**Maximum short axis diameter of fistula tracts (mm)**				
** At baseline**	4.21 (2.43)	4.65 (2.14)	5.13 (2.50)	5.17 (1.96)
** At the time of first remission achievement**	2.37 (2.85)	4.32 (2.37)	5.07 (2.87)	–
** At the time of relapse**	–	4.35 (2.48)	5.47 (2.61)	–
**Maximum short axis diameter of fistula tracts (%)^d^**				
** At the time of first remission achievement**	51.60 (52.42)	100.78 (41.74)	108.06 (48.96)	–
** At the time of relapse**	–	99.51 (42.28)	115.65 (40.05)	–

Data are mean (SD).

Abbreviations: LOCF, last observation carried forward; MRI, magnetic resonance imaging.

a Cases of remission at week 156 after relapse at week 52 were not included. One patient who discontinued after 831 days was included in the data because the efficacy result at the time of discontinuing from the study was combined remission, and the 831 days was included in the combined remission evaluation period at week 156.

b Data from relapsed fistula tracts only. For example, if a patient who relapsed had 2 fistula tracts and only 1 fistula tract relapsed, only the measurements from the relapsed tract are included in this column.

c Two patients who discontinued the study were included, and LOCF was adopted for 2 cases.

d Length at baseline defined as 100%.

The mean (SD) maximum short axis diameter of fistula tracts at baseline was shortest in patients who maintained combined remission until week 156 after achieving combined remission (4.21 [2.43] mm), followed by patients who relapsed during the 156-week period (4.65 [2.14] mm for all fistula tracts and 5.13 [2.50] mm for relapsed fistula tracts only), and those who did not achieve remission during the 156-week period (5.17 [1.96] mm) (Table 4). In patients who maintained combined remission until week 156 after achieving combined remission, the mean (SD) maximum short axis diameter of the fistula tract at the time of first achieving combined remission was 2.37 (2.85) mm, which was 51.60% of the value at baseline. On the other hand, in patients who relapsed during the 156-week period, the mean (SD) maximum short axis diameter of all fistula tracts at the time of first achieving combined remission was 4.32 (2.37) mm, which was barely changed from the value at baseline, at 100.78%. Representative MRI photographs from a patient who maintained combined remission and a patient who relapsed are shown in Figure S2.

At week 156, 8/15 patients (53.3%) in combined remission and 3/7 patients (42.9%) not in remission were receiving biologic treatment only (Table 5). The proportions of patients treated with biologics plus immunosuppressants at week 156 were 4/15 (26.7%) and 2/7 (28.6%) among patients in combined remission and non-remission, respectively. Among patients in combined remission, 8/15 patients (53.3%) had their medical treatment modified between week 52 and week 156, including 2 patients who had a dose increase of biologics or biologics plus immunosuppressants, 1 patient who changed to another biologic, 1 patient who started biologics, and 4 patients who discontinued either biologic or immunosuppressant therapy. By contrast, only 2/7 patients (28.6%) in non-remission experienced a change in medical treatment; both these patients discontinued either biologic or immunosuppressant therapy, and the patient on biologic therapy changed to another biologic.

**Table 5:** Medical treatment at week 156 (ITT population).^a^

Treatment	Medical treatment at week 156	
	Combined remission,^b^ *n* (%) (*N* = 15)	Non-remission,^b^ *n* (%) (*N* = 7)
**Biologics only**	8 (53.3)	3 (42.9)
**Immunosuppressants only**	1 (6.7)	0 (0.0)
**Biologics + immunosuppressants**	4 (26.7)	2 (28.6)
**Neither**	2 (13.3)	2 (28.6)
	
	**Change in medical treatment from weeks 52 to 156**	
**Modified treatment**	8 (53.3)	2 (28.6)
**No change**	7 (46.7)	5 (71.4)

Abbreviations: ITT, intent-to-treat; LOCF, last observation carried forward.

a LOCF was used for missing values.

b Combined remission status at week 156.

The treatment course was also evaluated for individual patients over the 156-week study period (Figure S3). There were 3 patients who achieved combined remission after relapse of the treated perianal fistula (Patients 2, 5, and 19). One of these patients (Patient 5) did not receive any concomitant medical treatment (biologics, immunosuppressants, or other) during the 156-week follow-up.

The number of patients in clinical remission and non-clinical remission at each evaluation visit is illustrated in Figure 3. At each evaluation visit from week 4 to week 156, there were changes in the numbers of patients in clinical remission, moving from non-clinical remission to clinical remission, moving from clinical remission to non-clinical remission, and in non-clinical remission.

**Figure 3:**
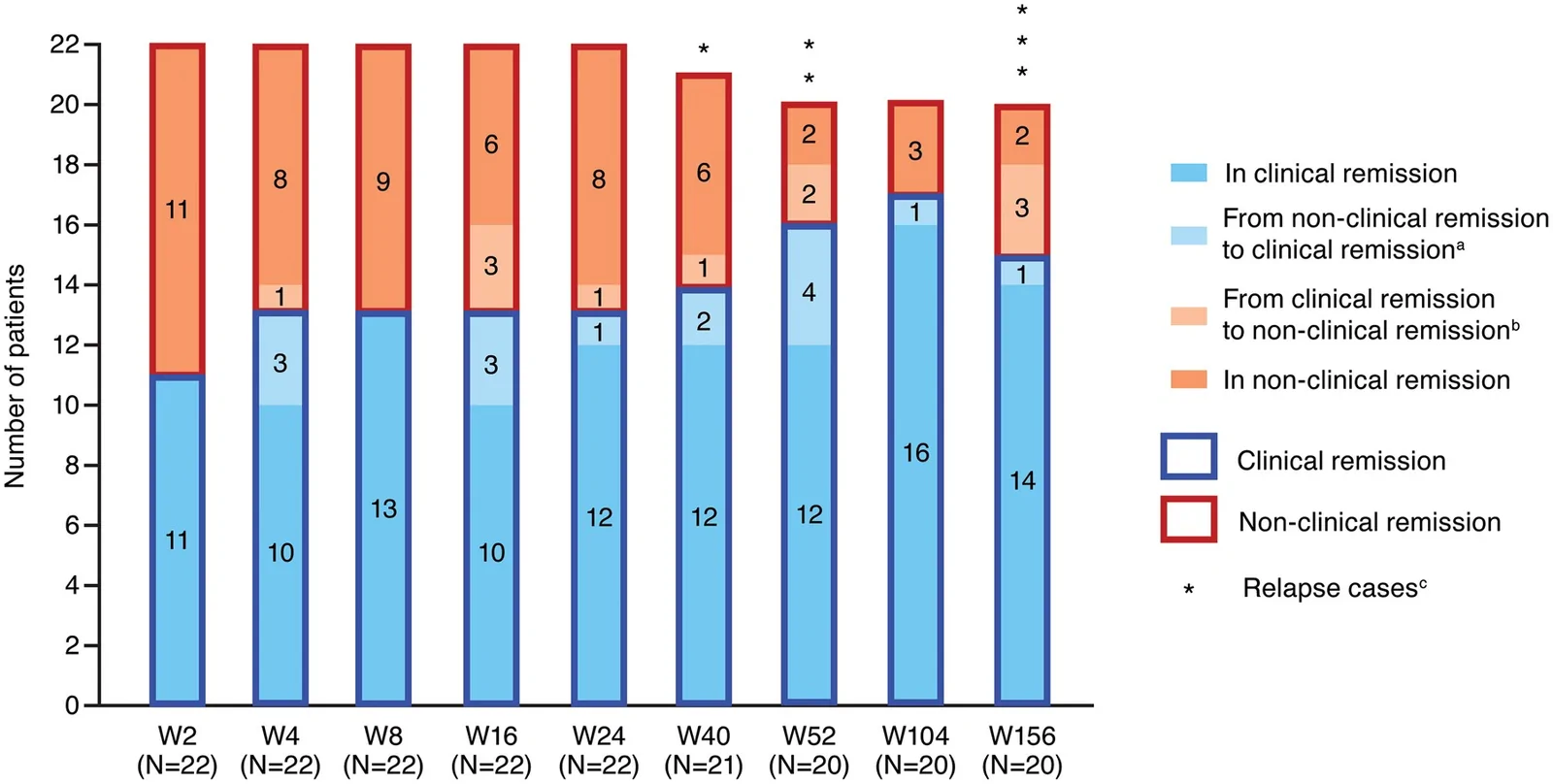
Number of patients in clinical remission and non-clinical remission at each evaluation visit (colored outlines), including the proportion of patients who transitioned from one state to the other (indicated by shading). Asterisks indicate each individual patient who relapsed at a given week. ^a^Non-clinical remission to clinical remission: Patients who were in non-clinical remission at the time of 1 previous evaluation visit but were in clinical remission at the time of evaluation (eg, non-clinical remission at 24 weeks, clinical remission at 52 weeks). ^b^Clinical remission to non-clinical remission: Patients who were in clinical remission at the time of 1 previous evaluation visit but were in non-clinical remission at the time of evaluation (eg, clinical remission at 24 weeks, non-clinical remission at 52 weeks). ^c^Relapse at weeks 40, 52, and 156 in patients with earlier combined remission. Relapse was defined as the clinically confirmed reopening of any of the treated external openings with active drainage, or the development of a collection >2 cm in the treated fistulas, confirmed by central MRI assessment. At weeks 40 and 52, relapse cases were in patients who achieved combined remission at week 24; at week 156, relapse cases were in patients who achieved combined remission at week 52. MRI, magnetic resonance imaging; W, week.

## Discussion

This study reports the 156-week follow-up results of darvadstrocel for complex perianal fistulas refractory to treatment in Japanese patients with CD. There were no new safety findings, and the safety profile of darvadstrocel through week 156 was comparable to that through week 52, as previously reported.^19^ Patients experienced sustained fistula closure, with combined and clinical remission rates at week 104 and week 156 being numerically similar to the previously reported combined and clinical remission rates at week 24 and week 52.^19^

Nine patients reported new serious TEAEs after week 52, including 1 patient with perianal fistula. However, none of these TEAEs were judged to be related to the study treatment. In the 52-week analysis of this clinical trial, 4 patients reported serious TEAEs, of which only 1 (CD) was considered treatment related.^19^ In the 104-week follow-up to the ADMIRE-CD trial of darvadstrocel, 4/40 patients had a new serious TEAE after 52 weeks of treatment (3 in the darvadstrocel group and 1 in the control group), and all serious TEAEs were related to perianal fistula or abscess.^20^ Although it is difficult to directly compare the results of the present study to the ADMIRE-CD trial due to the different follow-up periods (156 weeks vs 104 weeks), we note that the incidence of serious TEAEs related to perianal fistulas or abscess was numerically lower in our study compared with ADMIRE-CD.

The most common TEAEs through week 156 in descending order were perianal fistula, proctalgia, and nasopharyngitis; the same 3 TEAEs were also the most common during the first 52 weeks, although in a different order (proctalgia, nasopharyngitis, perianal fistula).^19^ Overall, 11/22 patients had perianal fistula reported as a TEAE and/or relapse during the 156-week follow-up period. Seven of these 11 patients had perianal fistula as a TEAE, comprising 4 patients who had worsening of treated perianal fistulas and 4 patients who had new perianal fistula (1 patient overlapped). Relapse was confirmed in 6 patients (2 of these cases overlap with perianal fistula as a TEAE). These results are consistent with the natural history of perianal fistula with CD, which often includes relapse of fistulas.^2^

Combined and clinical remission rates observed at week 104 and week 156 during follow-up were numerically similar to rates observed earlier in the trial. Combined remission rates were 59.1% (13/22 patients) and 68.2% (15/22 patients) at week 24 and week 52, respectively,^19^ and 77.3% (17/22) and 68.2% (15/22) at week 104 and week 156, respectively. Clinical remission rates were 72.7% (16/22 patients) at week 52,^19^ and 77.3% (17/22) and 68.2% (15/22) at week 104 and week 156, respectively. Both combined and clinical remission rates during the 156-week period increased numerically up to week 104 and then decreased slightly at week 156. Combined and clinical remission rates with darvadstrocel in ADMIRE-CD-I were, respectively, 51.5% and 55.3% at week 24, and 56.3% and 59.2% at week 52 (modified intent-to-treat population).^17^ The clinical remission rate in ADMIRE-CD-I patients included in a post-trial chart review was 53.5% at both 104 and 156 weeks after darvadstrocel administration.^22^ In ADMIRE-CD-II, combined and clinical remission rates with darvadstrocel were, respectively, 48.8% and 49.8% at week 24, and 41.0% and 43.1% at week 52.^21^ Differences in outcomes between the present study and the ADMIRE-CD trials may be attributable to differences in study design, procedures, patient characteristics, or concomitant therapies between the studies, among other factors.

In this long-term follow-up study, there were no restrictions on changes in concomitant medication after 52 weeks. Concomitant medication, especially biologics, controls intestinal lesions and may affect the outcome of surgical treatment of perianal fistulas.^12^ When we explored patterns of concomitant medication at week 156, we found that similar proportions of patients in remission and non-remission were treated only with biologics at this time (53.3% vs 42.9%, respectively). We also found that the proportion of patients who changed their concomitant medication after week 52 was higher in the remission group (8 patients; 53.3%) than in the non-remission group (2 patients; 28.6%). Four of the patients in the remission group had some form of treatment intensification from week 52 to week 156 (dose increase, change in biologic, or biologic initiation) in response to worsening intestinal lesions (3 patients) or pyoderma (1 patient). We hypothesize that intensifying treatment at the time of worsening of intestinal lesions helped maintain the combined remission of perianal fistulas in these patients. However, due to the small sample size of the study, we were unable to conclude whether changes in concomitant medication, particularly biologics, had a systematic effect on remission of perianal fistulas. In addition, when the treatment course was evaluated for individual patients over the 156 weeks following darvadstrocel administration, 3 patients achieved combined remission again after relapse. There are currently no data evaluating the survival and self-renewal of allogeneic adipose-derived stem cells at the injection site in humans, making it difficult to evaluate the long-term impact of darvadstrocel on remission after relapse. The use of systematic diagnostic imaging is critical for monitoring the therapeutic effect of darvadstrocel over both the short and long term. In future studies, combining diagnostic imaging with selective tissue or biomarker sampling would add to our understanding of long-term effects. We also note that in a patient who did not achieve remission by week 156, continued treatment with biologics led to significant improvement in perianal fistulas. These results suggest that it is important to control the pathology of CD by continuing medical treatment of the CD that is the cause of the perianal fistulas.

The efficacy of darvadstrocel in previous randomized controlled trials and clinical studies reported so far varies, including between-group differences with the placebo group.^21^^,^^23^^,^^24^ This may be due to the condition of the perianal fistula to which darvadstrocel is administered, suggesting that it is important to identify suitable cases. Treatments of perianal fistula with CD are often evaluated by assessing fistula closure clinically.^25^ However, MRI is increasingly being used to assess fistula healing, and MRI findings may be useful to identify fistulas that have closed externally but have an internal fistula tract.^25^ However, there is a lack of long-term studies of MRI findings compared with clinical assessment of fistula healing. To provide optimal treatment to patients with complex perianal fistulas, there is a need for more evidence, including MRI data from individual patients receiving darvadstrocel treatment. In this study, to explore predictors of remission and relapse, we additionally examined measurements obtained from MRI to identify specific factors that may be associated with the outcome of fistula healing. In patients who maintained combined remission until week 156 after achieving combined remission, the maximum short axis diameter of the fistula tract at the time of first achieving combined remission was approximately 50% of the value at baseline. By contrast, in patients who relapsed during the 156-week period, the mean maximum short axis diameter of the fistula tract at the time of first achieving combined remission did not change compared with the value at baseline. These data suggest that patients with a shorter maximum short axis diameter at the time of remission compared with baseline may be more likely to maintain remission, and this parameter may be useful as a predictor of prognosis. Furthermore, the mean (SD) length of fistula tract (distance between internal and external openings) at baseline was shortest in patients who maintained combined remission. However, the distance between internal openings and the anal verge and the degree of separation between anal sphincters did not appear to be useful as predictors of prognosis. The indicators objectively shown by MRI measurements are consistent with actual clinical experience and may serve as predictive factors. However, further detailed analysis is required to identify predictive factors.

At each evaluation visit during this study, there were changes in the proportions of patients in clinical remission, moving from non-clinical remission to clinical remission, moving from clinical remission to non-clinical remission, and in non-clinical remission. This pattern of treatment progress illustrates that perianal fistulas with CD are highly individualized, and appropriate treatment intervention is required according to the patient’s pathology and life events at the time. It is therefore important for surgeons and treating physicians to work together with other members of the multidisciplinary treatment team to provide better treatment and outcomes for patients with CD and perianal fistulas.

The strengths of this study include the multicenter design and 156-week follow-up period. In addition, the study included prospective follow-up of patients whereby healing (combined remission) was assessed using both clinical and MRI findings. A further strength is the insight that has been generated regarding possible predictors of remission and relapse using MRI data.

There are several limitations in this study. The study was single-arm and the number of patients was small; this likely affected the exploratory analyses the most (eg, Tables 4 and 5) and reduced the precision of estimates in the subgroup analyses. Therefore, the generalizability of the study results may be affected, and caution is required when interpreting the results, especially those of the subgroup analyses. However, the long follow-up and use of systematic MRI assessments partially offset the small sample size, given the depth of information provided. Patients’ QoL and cost-effectiveness specific to perianal fistula with CD were not evaluated, and these may be important for assessing the impact of darvadstrocel administration; for example, even if remission could not be achieved by administration of darvadstrocel, if the symptoms are reduced and patients’ QoL is improved compared with before treatment, it may be worthwhile to administer darvadstrocel. In future, studies using disease-specific questionnaires such as Crohn’s Anal Fistula QoL (CAF-QoL)^26^ will be needed. The CAF-QoL assesses disease-specific symptoms such as pain and discharge, the burden of fistula treatment, and the social and psychological impacts of fistulas.^26^ Furthermore, the collection of QoL data specific to perianal fistula with CD would make it possible to also assess cost-effectiveness in comparison with other therapies. Despite the good outcomes seen with darvadstrocel in the present study, a full evaluation of benefit is limited without these patient-reported measures and cost-effectiveness data.

## Conclusion

In this study of darvadstrocel administration for complex perianal fistulas, no new safety signals were identified during this 3-year follow-up period of the Japanese phase 3 study. These long-term data also suggest that combined and clinical remission of complex perianal fistulas is sustained in the long term (up to 156 weeks).

## Supplementary Material

otag090_Supplementary_Data

## Data Availability

The datasets, including the redacted study protocol, redacted statistical analysis plan, and individual participants data supporting the results reported in this article, will be made available within three months from initial request, to researchers who provide a methodologically sound proposal. The data will be provided after its de-identification, in compliance with applicable privacy laws, data protection, and requirements for consent and anonymization.
