# Supplementary material for: Long-term safety and efficacy of darvadstrocel for complex perianal fistulas in Japanese adults with Crohn’s disease: 3-year follow-up results
Source: Crohns Colitis 360. 2026 Aug 18;8(3):otag090. doi: 10.1093/crocol/otag090 (PMC13622351; doi:10.1093/crocol/otag090)
Supplement: Supplementary file 1 [file otag090_supplementary_data.docx]

SUPPLEMENTARY DATA

Long-Term Safety and Efficacy of Darvadstrocel for Complex Perianal Fistulas in Japanese Adults With Crohn's Disease: 3-Year Follow-up Results

**LIST OF TABLES AND FIGURES**

**Table S1**	Subgroup analyses: effect of age, sex, and number of internal/external openings on combined remission at week 104 and week 156.

**Figure S1**	Measurement of perianal fistula length from an MRI image.

**Figure S2**	Representative MRI photographs, with inflammatory fistula tracts identified by white arrows.

**Figure S3**	Treatment course by patient ID.

**Table S1.** Subgroup analyses: effect of age, sex, and number of internal/external openings on combined remission at week 104 and week 156.

|  | **Week 104** | | **Week 156** | |  |
| --- | --- | --- | --- | --- | --- |
|  | **Remission** | **Non-remission** | **Remission** | **Non-remission** | **Total, n** |
| Age |  |  |  |  |  |
| ≤65 years | 17 (77.3) | 5 (22.7) | 15 (68.2) | 7 (31.8) | 22 |
| >65 years | 0 | 0 | 0 | 0 | 0 |
| Sex |  |  |  |  |  |
| Male | 11 (78.6) | 3 (21.4) | 9 (64.3) | 5 (35.7) | 14 |
| Female | 6 (75.0) | 2 (25.0) | 6 (75.0) | 2 (25.0) | 8 |
| IOs at baseline |  |  |  |  |  |
| 1 IO | 13 (81.3) | 3 (18.8) | 11 (68.8) | 5 (31.3) | 16 |
| 2 IOs | 4 (66.7) | 2 (33.3) | 4 (66.7) | 2 (33.3) | 6 |
| EOs at baseline |  |  |  |  |  |
| 1 EO | 3 (100) | 0 | 2 (66.7) | 1 (33.3) | 3 |
| 2 EOs | 11 (78.6) | 3 (21.4) | 10 (71.4) | 4 (28.6) | 14 |
| 3 EOs | 3 (60.0) | 2 (40.0) | 3 (60.0) | 2 (40.0) | 5 |
| IOs/EOs at baseline |  |  |  |  |  |
| 1 IO + 1 EO | 3 (100) | 0 | 2 (66.7) | 1 (33.3) | 3 |
| 1 IO + ≤2 EOs | 10 (76.9) | 3 (23.1) | 9 (69.2) | 4 (30.8) | 13 |
| 2 IOs + ≤2 EOs | 4 (66.7) | 2 (33.3) | 4 (66.7) | 2 (33.3) | 6 |

All data are n (%) unless specified otherwise.

EO, external opening; IO, internal opening.

**Figure S1.** Measurement of perianal fistula length from an MRI image. On a T2-weighted coronal image parallel to the course of the perianal fistula, the distance between the internal and external openings was measured. In this example, because the course is curved, the sum of 2 straight lines was used to calculate the full fistula length (red line).

MRI, magnetic resonance imaging.

**Figure S2.** Representative MRI photographs, with inflammatory fistula tracts identified by white arrows. (A) Baseline image from Patient A, who maintained combined remission until week 156, showing 2 fistula tracts. (B) Baseline image from Patient B, who relapsed at week 156.
(C) Post-treatment image at week 52 from Patient A, showing that both fistula tracts had reduced in size. (D) Post-treatment image at week 156 from Patient A, showing that the size reduction for both fistula tracts was maintained.

**Patient A:** Female; duration of CD 7.67 years; treatment of CD, infliximab and azathioprine; number of perianal fistulas, 1 IO and 2 EOs; mean length of 2 fistula tracts at baseline was 17.8 mm and 22.9 mm; maintained combined remission until week 156; mean maximum short axis diameter of 2 fistula tracts was 3.6 mm and 3.2 mm at baseline, and 1.4 mm and 1.3 mm at the time of first achieving combined remission. There was no change in medical treatment through week 156.

**Patient B:** Male; duration of CD 6.63 years; treatment of CD, infliximab and azathioprine; number of perianal fistulas, 1 IO and 2 EOs; mean length of 2 fistula tracts at baseline was 40.5 mm and 51.7 mm; combined remission at week 24 and then fistula #2 relapsed at week 156; mean maximum short axis diameter of 2 fistula tracts was 3.5 mm (fistula #1) and 9.1 mm (fistula #2) at baseline, 3.8 mm (fistula #1) and 7.1 mm (fistula #2) at the time of first achieving combined remission, and 1.9 mm (fistula #1) and 8.1 mm (fistula #2) at the time of relapse. Medical treatment was changed after week 52: azathioprine was discontinued 689 days after darvadstrocel administration due to the worsening of intestinal lesions and side effects but was resumed 963 days later due to the worsening of intestinal lesions.

CD, Crohn’s disease; EO, external opening; IO, internal opening; MRI, magnetic resonance imaging.

**Figure S3.** Treatment course by patient ID. For each patient, the top row shows efficacy evaluation results, and the bottom row (if present) shows TEAEs reported as perianal fistula. Orange boxes indicate non-remission or relapse. Green boxes indicate response, defined as clinically confirmed closure of more than half of the treated external openings despite gentle finger compression. Blue boxes indicate clinical or combined remission. Grey boxes indicate patients who discontinued the study.

^a^This patient is a case of relapse because an abscess >2 cm was observed, but also a case of clinical remission because there was no drainage.

^b^Although the patient dropped out at 831 days, the treated anal fistula achieved combined remission in the evaluation at the time of dropout, and because 831 days was included in the 156-week evaluation period, the patient was counted as a patient in combined remission at week 156.

Clin, clinical remission; Comb, combined remission; d, day; NR, non-remission; PF, perianal fistula; Rel, relapse; Resp, response; TEAE, treatment-emergent adverse event; W, Week.

| ID | W2 | W4 | W8 | W16 | W24 | W40 | W52 | W104 | W156 |
| --- | --- | --- | --- | --- | --- | --- | --- | --- | --- |
| 1 | NR | NR | NR | NR | NR | NR | NR | NR | Resp |
|  |  |  |  |  |  |  | Worsening of treated PF (579 d onwards) | | |
| 2 | Clin | Clin | Clin | Clin | Comb | Clin | Rel | Rel | Comb |
|  |  |  |  |  |  |  | Worsening of treated PF (W52) | |  |
| 3 | Clin | Clin | Clin | Clin | Comb | Clin | Comb | Comb | Comb |
| 4 | Clin | Clin | Clin | Clin | Comb | Clin | Comb | Comb | Comb |
| 5 | Clin | Clin | Clin | Clin | Comb | Clin | Clin and **Rel**^a^ | Comb | Comb |
|  |  | New PF (28-161 d) | |  |  | Worsening of treated PF (335-455 d) | |  |  |
| 6 | Clin | Clin | Clin | Clin | Comb | Clin | Comb | Comb | Comb |
| 7 | Clin | Clin | Clin | Clin | Comb | Clin | Comb | Comb | Comb |
|  | New PF (16 d onwards) | |  |  |  |  |  |  |  |
| 8 | Resp | Resp | Resp | Resp | Resp | Resp | Comb | Comb | Dropout/Comb^b^ |
|  |  |  |  |  |  |  |  |  | New PF (792 d onwards) |
| 9 | Resp | NR | NR | Clin | NR | Dropout |  |  |  |
|  |  |  |  | Worsening of treated PF (116 d onwards) | | |  |  |  |
| 10 | NR | NR | NR | Clin | Comb | Clin | Comb | Comb | Comb |
|  |  |  |  |  |  |  |  |  | New PF (982-1088 d) |
| 11 | NR | Clin | Clin | Resp | Resp | Resp | Dropout |  |  |
| 12 | NR | NR | NR | Clin | Comb | Clin | Comb | Comb | Comb |
| 13 | Resp | Resp | NR | Resp | Resp | Resp | Comb | Comb | Rel |
| 14 | NR | NR | NR | NR | NR | Clin | Comb | Comb | Rel |
| 15 | NR | NR | Resp | Resp | Resp | Resp | Resp | Resp | Resp |
| 16 | NR | Clin | Clin | Resp | Comb | Clin | Comb | Comb | Comb |
| 17 | Clin | Resp | NR | Resp | Resp | Clin | Resp | Comb | Comb |
| 18 | Resp | Clin | Clin | Clin | Comb | Clin | Comb | Comb | Rel |
| 19 | Clin | Clin | Clin | Clin | Comb | Rel | Comb | Comb | Comb |
| 20 | Clin | Clin | Clin | Clin | Comb | Clin | Comb | Comb | Comb |
| 21 | Clin | Clin | Clin | Clin | Comb | Clin | Comb | Comb | Comb |
| 22 | Clin | Clin | Clin | Resp | Resp | Resp | Comb | Comb | Comb |
